# Contrasting Patterns of *Asaia* Association with Pyrethroid Resistance Escalation between the Malaria Vectors *Anopheles funestus* and *Anopheles gambiae*

**DOI:** 10.3390/microorganisms11030644

**Published:** 2023-03-02

**Authors:** Fleuriane Metissa Djondji Kamga, Leon M. J. Mugenzi, Magellan Tchouakui, Maurice Marcel Sandeu, Claudine Grace Tatsinkou Maffo, Maximilienne Ascension Nyegue, Charles S. Wondji

**Affiliations:** 1 Centre for Research in Infectious Diseases (CRID), Yaoundé P.O. Box 13591, Cameroon; 2 Department of Microbiology, Faculty of Science, University of Yaoundé I, Yaoundé P.O. Box 812, Cameroon; 3 Department of Microbiology and Infectious Diseases, School of Veterinary Medicine and Sciences, University of Ngaoundéré, Ngaoundéré P.O. Box 454, Cameroon; 4 Vector Biology Department, Liverpool School of Tropical Medicine, Liverpool L3 5QA, UK

**Keywords:** malaria, *Anopheles gambiae*, *Anopheles funestus*, *Asaia* spp., resistance escalation, cytochrome P450, voltage-gated sodium channel

## Abstract

Microbiome composition has been associated with insecticide resistance in malaria vectors. However, the contribution of major symbionts to the increasingly reported resistance escalation remains unclear. This study explores the possible association of a specific endosymbiont, *Asaia* spp., with elevated levels of pyrethroid resistance driven by cytochrome P450s enzymes and voltage-gated sodium channel mutations in *Anopheles funestus* and *Anopheles gambiae.* Molecular assays were used to detect the symbiont and resistance markers (*CYP6P9a/b*, *6.5 kb*, *L1014F*, and *N1575Y*). Overall, genotyping of key mutations revealed an association with the resistance phenotype. The prevalence of *Asaia* spp. in the FUMOZ_X_FANG strain was associated with the resistance phenotype at a 5X dose of deltamethrin (OR = 25.7; *p* = 0.002). Mosquitoes with the resistant allele for the markers tested were significantly more infected with *Asaia* compared to those possessing the susceptible allele. Furthermore, the abundance correlated with the resistance phenotype at 1X concentration of deltamethrin (*p* = 0.02, Mann-Whitney test). However, for the MANGOUM_X_KISUMU strain, findings rather revealed an association between *Asaia* load and the susceptible phenotype (*p* = 0.04, Mann-Whitney test), demonstrating a negative link between the symbiont and permethrin resistance. These bacteria should be further investigated to establish its interactions with other resistance mechanisms and cross-resistance with other insecticide classes.

## 1. Introduction

Malaria remains a major public health burden in the tropical world, particularly in Africa, where >90% of malaria cases and mortality have been reported [1]. Malaria prevention has recently been boosted by the adoption of the first vaccine in 2021 [2]. However, vector control through the use of insecticide-based interventions such as long-lasting insecticidal nets (LLINs) and indoor residual spraying (IRS) remains the cornerstone of malaria control and elimination programs [3]. Even so, increasing reports of elevated levels of pyrethroid resistance in the major vectors, including *Anopheles gambiae* and *Anopheles funestus,* are threatening to derail the efficacy of these control tools [4]. In several countries, including Malawi [5], Uganda [6], and Ghana [7], *An. funetus* and *An. gambiae* populations were both reported to exhibit elevated levels of insecticide resistance, surviving exposure to not only 10X the discriminating concentration (DC) of pyrethroids but also piperonyl butoxide (PBO) plus pyrethroid-treated nets [6]. Similarly, in Burkina Faso, a considerable reduction in the efficacy of the treated nets was observed with *An. gambiae* [8] and recently in Mozambique against *An. funestus* s.s. [9]. The causal mechanisms underlying this phenomenon of resistance escalation remain uncharacterized and could be multifactorial. The common insecticide resistance mechanisms are metabolic resistance, notably through the overexpression of detoxification enzymes including cytochrome P450s, modification of the insecticide target site (voltage-gated sodium channel), and thickening of the cuticle [10,11]. In addition, constituents of the *Anopheles* microbiota have recently emerged as contributing factors to insecticide resistance [12,13,14,15].

Arthropods, like almost all other invertebrates, live in symbiosis with their microbiota, which considerably influences their life traits: fitness, nutrient supply, protection against pathogens through immune system stimulation, development, and synthesis of specific toxins [16]. Mosquito microbiota could be defined as a heterogeneous and complex environment consisting of bacteria, fungi, viruses, parasites, and other eukaryotic cells that stably or transiently infect the cuticle and internal organs. Their composition in *Anopheles* species has been studied mainly by culturing or sequencing 16S rRNA. Although the composition of this microbiota is dynamic and variable depending on the *Anopheles* species, an overview provided by Gendrin and Christophides (2013) reported the identification of approximately 41 bacterial genera in ten *Anopheles* species, with nine being highly abundant. These include *Pseudomonas, Aeromonas*, *Asaia*, *Comamonas*, *Elizabethkingia*, *Enterobacter*, *Klebsiella*, *Pantoea,* and *Serratia* [17]. One interesting aspect of the *Anopheles* microbiota is its negative influence on *Plasmodium* development within mosquitoes [18,19]. For instance, it has been shown that bacterial growth after a blood meal causes the synthesis of antimicrobial peptides and other immune effectors that have antiparasitic properties [20]. In addition, an *Enterobacter* strain (EspZ) isolated from wild *Anopheles arabiensis* mosquitoes was found to directly inhibit *Plasmodium* development via elevated synthesis of reactive oxygen species (ROS) [19].

These findings also make it plausible that a link could exist between microorganisms and the resistance of these mosquitoes to different classes of insecticide [21]. Recent studies have highlighted a link between insecticide resistance and the bacterial symbionts of arthropods. For example, in agriculture, this notion has been clearly described for the bean bug *Riptortus pedestris,* where *Burkholderia* bacteria were shown to establish a specific and beneficial symbiosis with the bugs, conferring resistance to fenitrothion (organophosphate) [22]. Similarly, in the moth *Plutella xylostella*, bacteria have been shown to influence some key biological traits of their hosts. *Enterococcus* spp. increased their resistance to chlorpyrifos (organophosphate), whereas that of *Serratia* spp. was rather reduced [23]. With the advent of new molecular tools, research in this field has extended to pathogen vectors such as *Anopheles*. Dada et al., (2018), using whole-metagenome sequencing of the microbiota of fenitrothion-sensitive and resistant strains of *Anopheles albimanus,* revealed a significant enrichment of organophosphate-degrading enzymes but also an overabundance of *Klebsiella* spp. in the resistant populations. It has been suggested that a possible selection of these bacteria may have occurred in parallel with the resistance phenotype in response to previous insecticide exposure [24]. Furthermore, in metagenomic studies carried out by Dada et al., (2019) on the F_1_ offspring of pyrethroid-resistant mosquitoes showed a significant increase in bacteria of the genus *Pseudomonas* and *Pantoea* species known to metabolize pyrethroids [14]. Similarly, a study conducted by Omoke in 2021, reported the significant presence of the bacterial taxa *Sphingobacterium*, *Lysinibacillus*, and *Streptococcus* in populations of *An. gambiae* that are resistant to 5X the standard dose of permethrin [12].

Recently, phylogenetic analyses of *Asaia* spp. Sequences, isolated from *Ceratitis capitata,* revealed that some strains have an ancestral pyrethroid hydrolase gene that could affect pyrethroid insecticide metabolism [25]. In *Anopheles coluzzii* species, a significant enrichment of *Asaia* bacteria was reported in susceptible mosquitoes compared to resistant mosquitoes, which highlights that the contribution of this symbiont in determining the resistance phenotype is very complex and host-specific [13]. This controversial role of *Asaia* spp., in pyrethroid resistance makes it an interesting target, especially as it is one of the best-characterized symbionts. *Asaia* spp. which belongs to the Acetobacteraceae family, was isolated for the first time from the *Anopheles* species. It is a gram-negative, aerobic, rod-shaped bacterium that is transmitted vertically from parent to offspring [26] and horizontally from one mosquito to another during feeding or copulation [27,28]. This enables it to form stable and long-term associations with its hosts over several generations. It can be easily cultured and transformed with exogenous DNA [26]. Considering the hypothesis that mosquito microbiota is affected by insecticide exposure, this study was conducted to comparatively characterize any association between the *Asaia* community and the escalation of pyrethroid resistance observed in *An. funestus* and *An. gambiae* species. These findings revealed a statistical association between the prevalence and abundance of this symbiont and deltamethrin-resistant mosquitoes in the *An. funestus* species. Furthermore, a negative association between the symbiont and permethrin resistance was observed in the *An. gambiae* strain.

## 2. Materials and Methods

### 2.1. Mosquito Strains and Rearing

This study was carried out using the major malaria vectors, *An. funestus* and *An. gambiae.* Given that the already characterized major insecticide resistance markers are almost fixed in the field mosquito populations, this hinders the possibility of clearly linking the genotypes to the presence of a particular bacterium. To overcome this issue, two reciprocal crosses between resistant and susceptible mosquitoes were established at the insectarium of the Center for Research in Infectious Diseases (CRID). For *An. funestus*, the fully susceptible strain FANG (homozygous susceptible to all resistance markers) colonized from southern Angola [29] was crossed with the fully resistant strain FUMOZ-R (homozygous resistant to some cytochrome P450 markers) originating from Mozambique [30]. For *An. gambiae*, crossings between KISUMU (the susceptible strain) [31] and field-resistant mosquitoes collected from MANGOUM (an agricultural setting located in the western region of Cameroon) [32] were established.

To commence the crossing, one hundred pupae of each strain were collected and individually placed in hemolysis tubes for the adults to emerge. Fifty females of the resistant colony were mixed in the same cage (30 cm × 30 cm) with fifty males of the susceptible mosquito colony (and reciprocally) for random mating. Females were allowed to lay eggs, and larvae were reared and fed with Tetramin^®^ baby fish food until their emergence as adult mosquitoes in the first generation (F1). The generated hybrids were reared until the fourth progeny (F4) under standard insectary conditions of 27 ± 2 °C and 80  ±  10% relative humidity.

### 2.2. Evaluating the Susceptibility Profile of An. funestus and An. gambiae Strains to Pyrethroids

Susceptibility assays against pyrethroids were performed with the parental lines: FUMOZ (N = 193, 8 replicates), MANGOUM (N = 160, 8 replicates), and the *An. funestus* F4 hybrid strains (N = 257, 10 replicates) as well as the *An. gambiae* hybrid strains (N = 232, 10 replicates) following the WHO protocol [33]. They were exposed to type I pyrethroid (permethrin (0.75%)) and type II pyrethroid (deltamethrin (0.05%)). Twenty to twenty-five females (3 to 5 days old) were aspirated and transferred into holding tubes for 1 h, then introduced into tubes containing insecticide-treated papers. Control tubes with non-impregnated papers were prepared in parallel for each bioassay. After 1 h of exposure, the knockdown effect was scored, and mosquitoes were transferred into holding tubes. Mortality was recorded 24 h post-exposure. The bioassay room conditions were set up to 27 ± 2 °C and 75 ± 10% relative humidity. The guidelines for the interpretation of bioassays were established based on the WHO insecticide susceptibility testing guidelines. “Resistance” and “Susceptible” phenotypes were confirmed by mortalities below 90% and greater than 98%, respectively [33]. Based on the mortality rate observed at the diagnostic dose (1X), a total of 1311 *An. funestus* (58 replicates) and 1410 *An. gambiae* (64 replicates) hybrid mosquitoes were used to evaluate the intensity of resistance to pyrethroids using dose-response assays (5X and 10X) and the time-course exposure method. According to the WHO guidelines, we confirmed “low intensity of resistance” with a mortality rate of mosquitoes of more than 98% at 5X the diagnostic concentration of insecticide. “Moderate intensity of resistance” was considered with mortality rates below 98% at 5X and greater than 98% at 10X concentrations of insecticide. “High intensity of resistance” was therefore confirmed with a mortality rate superior to 98% at 10X concentration of the insecticide [33]. Depending on the phenotype, samples were stored in silica gel at −20 °C for molecular analysis.

### 2.3. Assessing the Contribution of Known Molecular Markers to the Escalation of Pyrethroid Resistance in An. funestus and An. gambiae

Before evaluating the role of *Asaia* in the escalation of resistance in the above strains, we first assessed the contribution of the recently detected cytochrome P450 markers (*CYP6P9a*, *CYP6P9b,* and 6.5kb-SV) and the target site mutations (L1014F and N1575Y).

#### 2.3.1. Contribution of CYP6P9a, CYP6P9b, and 6.5 kb-SV Mutations to the Escalation of Pyrethroid Resistance in *An. funestus*

The individual mosquitoes (alive and dead) of hybrid *An. funestus* (F4) were sterilized with 70% ethanol and rinsed twice with 1X PBS (Phosphate Buffer Solution) to remove non-attached bacteria, thus preventing sample contamination. Genomic DNA was extracted following the Livak protocol [34], and the respective cytochrome P450 markers were genotyped using established protocols. *CYP6P9a* and *CYP6P9b* were genotyped using RFLP-PCR [35,36]. The primers listed in Table S1 were used in the first step of the amplification of the partial *CYP6P9a* and *CYP6P9b* upstream regions. DNA amplicons were visualized on 2.0% agarose gel stained with Midori Green, and the results were interpreted based on the observed band sizes. For the second step, the TaqI enzyme (restriction site (5′-TCGA-3′)) and *Tsp*45I enzyme (restriction site (5′-GTSAC-3′)) were used to digest the PCR products to detect the CYP6P9a_R and CYP6P9b_R resistant alleles, respectively, as previously described [35,36]. The structural variation of *6.5 kb-SV* was also detected using the PCR method [37]. Three primers flanking the insertion region (FG_5F, FG_3R, and FZ_INS5R) were used for genotyping the mutation (Table S1). The PCR products were resolved on a 1.5% agarose gel stained with Midori Green and visualized on a UV transilluminator. Odds ratios and Fisher’s exact tests were used to establish the statistical significance of any association between these pyrethroid resistance markers and the ability of mosquitoes to survive increasing concentrations of insecticides.

#### 2.3.2. Contribution of the Voltage-Gated Sodium Channel (VGSC) Mutations, L1014F and N1575Y, to the Escalation of Pyrethroid Resistance in *An. gambiae*

Genomic DNA of hybrid *An. gambiae* (MANGOUM_X_KISUMU F4) mosquitoes was extracted as described above and used to genotype the L1014F and N1575Y voltage-gated sodium channel (VGSC) mutations via TaqMan SNP genotyping assays [38]. Probes combined with primers were differentiated using two specific fluorescent dyes, FAM corresponding to homozygous-resistant genotypes (RR) and HEX to homozygous-susceptible (SS). The sequences of primers used for these methods are given in Table S1. The statistical test to evaluate association was performed as described above.

### 2.4. Assessing the Contribution of Asaia spp. in the Escalation of Pyrethroid Resistance in An. funestus and An. gambiae Mosquitoes

In addition to host-mediated resistance mechanisms, the role of *Asaia* spp. in insecticide resistance was investigated. First, we determined the statistical association between the prevalence of *Asaia* spp. and the resistance phenotype/genotype. Thereafter, we checked for any correlation between the abundance of the bacteria and the resistance phenotype/genotype.

#### 2.4.1. Detection of *Asaia* spp. Frequency in Anopheles Strains

The presence of *Asaia* spp. was determined via a conventional PCR assay using specific primers (16S RNA_F and Asarev_R) that amplify 676 base pairs (bp) of a partial sequence of the *Asaia* 16S rRNA gene [39]. PCR products were visualized on a 1.5% agarose gel stained with Midori green dye using a UV transilluminator. Samples of the expected size were selected randomly and purified using Exonuclease-I (Exo I) and Shrimp Alkaline Phosphatase (Exo-SAP protocol) according to the New England Biolabs protocol (NEB, Ipswich, MA, USA), which involved incubation at 37 °C for 10 min and 80 °C for 20 min. Purified amplicons were sequenced by Microsynth Seqlab GmbH company (Göttingen city, Germany) and analyzed using BLASTn in NCBI (www.ncbi.nlm.nih.gov/blast/; accessed on 17 March 2022) to confirm effective amplification of the target *Asaia* spp. gene. Odds ratios and Fisher exact tests were the statistical tests used to assess the association between the prevalence of the symbiont and the resistant phenotype/genotype.

#### 2.4.2. Quantification of *Asaia* spp. in *Anopheles* Strain

The abundance of *Asaia* was assessed in duplicate using quantitative polymerase chain reaction (qPCR). The AsaH1_F and Asar_R primers were used to quantify the symbiont in each DNA template (Table S1). qPCR of Ribosomal Protein S7 (single copy gene) of both species (VectorBase ID: AFUN007153 in *An. funestus*; orthologous to *An. gambiae*: AGAP010592) was performed in parallel to normalize the *Asaia* load (Table S1). The reactions were run in a total volume of 10 μL, containing 5 μL of 2X Brilliant II SYBR^®^ Green QPCR Master Mix (Agilent), 1 µM of each primer, 1 μL of nuclease-free water, and 2 μL template DNA. The reactions were run on an MX3005 real-time PCR system (Agilent) following a dissociation curve (95 °C for 10 s, 65 °C for 60 s, and 97 °C for 1 s [40]. The standard curve of each gene was generated by diluting one-tenth of the DNA extracted from *Asaia* cultures and the purified PCR amplicons of the ribosomal protein S7 gene. Ct values of >35 were considered uninfected or undetectable. The normalized amount of bacterial DNA was estimated by the relative ratio of *Asaia* gene copy numbers to RSP-7 gene copy numbers. Mann Whitney and Kruskal Wallis non-parametric tests were used to compare the load of *Asaia* spp. between phenotype (alive and dead) and genotype, respectively, via GraphPad Prism 8.2.0.

## 3. Results

### 3.1. Resistance Profile of Anopheles Funestus and Anopheles gambiae Strains

**Figure 1:**
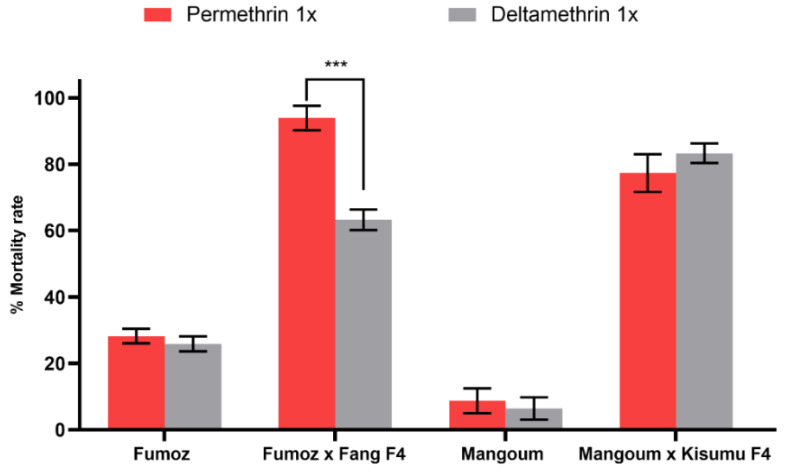
Susceptibility profile of *An. funestus* and *An. gambiae* strains to 1X DC of permethrin and deltamethrin. Results are the average of recorded mortalities following 60 min of exposure. Data are shown as mean ± SEM. *** *p* < 0.0001.

The laboratory-resistant strain FUMOZ experienced low mortality rates when exposed to permethrin (1X: 27.6 ± 1.1%) and deltamethrin (1X: 25.9 ± 2.2%). The F4 progeny of hybrid strain FUMOZ_X_FANG showed possible resistance to permethrin (1X: 93.9 ± 3.7%), but a persistent level of resistance to deltamethrin (1X: 63.3 ± 3.1%), suggesting a greater level of resistance to type II pyrethroids. In the MANGOUM field mosquitoes, the resistance pattern to the two insecticides was as follows: permethrin (1X: 8.7 ± 3.7%) and deltamethrin (1X: 6.4 ± 3.3), and the crossing MANGOUM_X_KISUMU F4 progeny exhibited a higher mortality rate to permethrin (1X: 77.4 ± 5.6%) and deltamethrin (1X: 83.3 ± 2.9%) (Figure 1).

**Figure 2:**
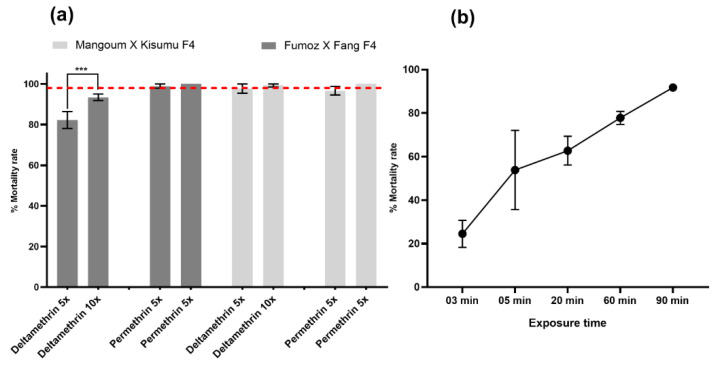
Resistance intensity of the *An. funestus* and *An. gambiae* hybrid strains to pyrethroids. (**a**) Estimation of resistance intensity with 5X and 10X the diagnostic concentrations of permethrin and deltamethrin in both *Anopheles* mosquitoes. (**b**) Time-point mortality rates to permethrin 1X DC of *An. gambiae* hybrid mosquitoes to generate the highly resistant (HR: alive 90 min) and highly susceptible (HS: dead 03 min). Data are shown as mean ± SEM. *** *p* < 0.0001.

Due to the resistance status of *An. funestus* hybrid strains to permethrin and deltamethrin, we conducted intensity bioassays with 5X and 10X concentrations of these insecticides. A high intensity of resistance to deltamethrin was observed at 5X (82.3 ± 4.2%) and 10X (93.4 ± 1.6%) doses with the F4 progeny of the FUMOZ_X_FANG hybrids. A significant increase in mortality was detected in mosquitoes exposed to deltamethrin 5X compared to 10X (χ^2^ = 20.4, *p* < 0.0001). However, for the same strain, we observed a low-intensity resistance to permethrin 5X and 10X, with mortality rates of 98.9 ± 1.1% and 100%, respectively. The MANGOUM_X_KISUMU F4 hybrid (*An. gambiae*) mosquitoes displayed a moderate intensity of resistance to all pyrethroids tested. This was observed with mortality rates of 96.7 ± 2.1% and 100% for permethrin 5X and 10X, respectively, and 97.7 ± 2.3% for deltamethrin 5X and 99.3 ± 0.7% for deltamethrin 10X (Figure 2). Regarding the profile we observed for the MANGOUM_X_KISUMU hybrid mosquitoes, they were more resistant to permethrin when compared to deltamethrin. For this reason, a time-course exposure method was used to select highly permethrin-resistant (HR) and highly susceptible (HS) phenotypes. HR mosquitoes were selected with a 90 min exposure time (mortality rate = 91.6 ± 1.5%), whereas HS mosquitoes were selected at a 3 min exposure time (mortality rate = 24.5 ± 10.7%) (Figure 2).

### 3.2. Contribution of Cytochrome P450 Markers in the Escalation of Deltamethrin Resistance in An. funestus

**Figure 3:**
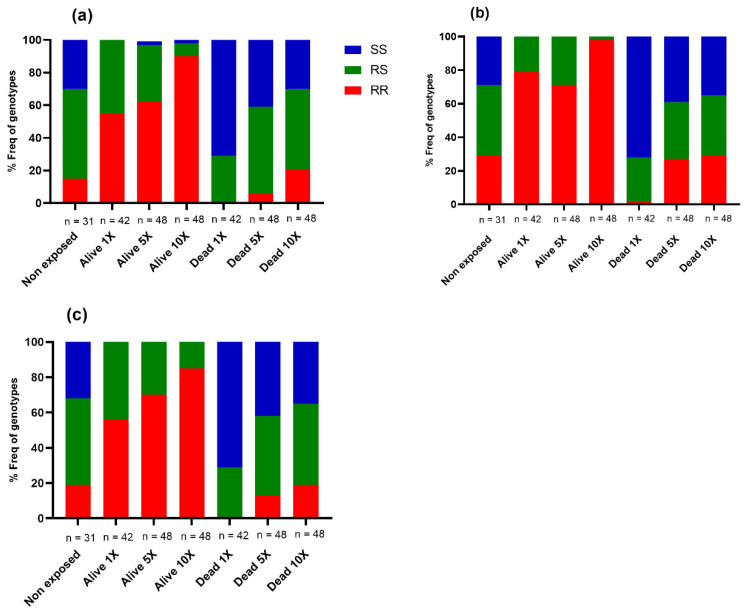
Distribution of the proportions of the cytochrome P450 genotypes between dead and alive in F4 of *An. funestus* hybrid mosquitoes after WHO bioassays with 1X, 5X, and 10X concentrations of deltamethrin. (**a**) distribution of the *CYP6P9a* genotypes, (**b**) repartition of the *CYP6P9b* genotypes, and (**c**). 6.5 kb insertion genotypes. RR represents the proportion of individuals harboring the homozygote-resistant mutation. SS corresponds to the proportion of individuals with the homozygote susceptible genotype, and RS represents the frequency of heterozygote mosquitoes. 1X: Diagnostic concentration of the insecticide; 5X and 10X correspond to 5 and 10 times the diagnostic concentration of insecticide, respectively.

In total, 309 DNA samples (Alive = 138, Dead = 140, Non-exposed = 31) were genotyped for *CYP6P9a*, *CYP6P9b*, and 6.5-kb insertion. The proportion of each genotype is presented in Figure 3 below.

A significant difference in the distribution of genotypes of each mutation was observed between dead and alive mosquitoes after exposure to 1X deltamethrin (*CYP6P9a*: χ^2^: 54.58, *p* < 0.0001; *CYP6P9b*: χ^2^: 60.32, *p* < 0.0001; 6.5 kb-SV: χ^2^: 52.82, *p* < 0.0001). The frequency of homozygote resistant (RR) and heterozygote resistant (RS) of each marker was higher in the alive group compared to the dead group. Assessment of the odds ratio (OR) revealed a stronger association between the resistant allele of each marker and the ability of the mosquitoes to survive exposure to deltamethrin, supporting the involvement of these alleles in the observed resistance (Table S2). Concerning the increased intensity of resistance, a significant difference was found in the distribution of genotypes between alive and dead mosquitoes exposed to 5X DC of deltamethrin (*CYP6P9a/*χ^2^: 39.81, *p* < 0.0001; *CYP6P9b/*χ^2^: 25.01, *p* < 0.0001; *6.5 kb/*χ^2^: 38.72, *p* < 0.0001). The same pattern was observed with 10X the concentration (*CYP6P9a/*χ^2^: 42.27, *p* < 0.0001; *CYP6P9b/*χ^2^: 39.85%, *p* < 0.0001; *6.5 kb/*χ^2^: 45.24, *p* < 0.0001). Mosquitoes possessing the resistant allele had a greater ability to survive exposure to 5X and 10X DC of deltamethrin compared to those with the susceptible allele, indicating that these markers are associated with the escalation of deltamethrin resistance in *An. funestus* (Table S2).

**Figure 4:**
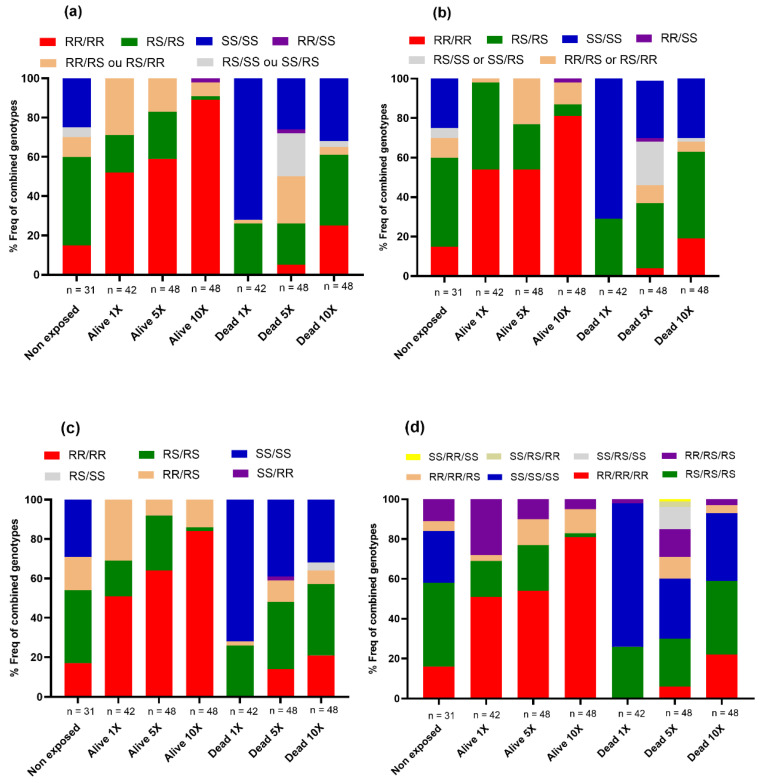
Distribution of combined genotypes of cytochrome P450 mutations between alive and dead after exposure to pyrethroids. (**a**) CYP6P9a and CYP6P9b association; (**b**) 6.5 kb-SV and CYP6P9a; (**c**) 6.5 kb-SV and CYP6P9b combination; (**d**) triple combination: 6.5 kb-SV, CYP6P9a, and CYP6P9b.

Looking at the cumulative impact of these markers, Figure 4 depicts the distribution of genotypes of two combined markers (*CYP6P9a/CYP6P9b; CYP6P9a/6.5 kb; CYP6P9b/6.5 kb*) and three markers (*CYP6P9a/CYP6P9b/6.5 kb*). We observed that mosquitoes harboring a combination of double- or triple homozygote-resistant genotypes (for example, *CYP6P9a* (RR)/*CYP6P9b* (RR) or *CYP6P9a* (RR)/*CYP6P9b* (RR)/6.5 kb (RR)) were significantly more likely to survive exposure to the different doses of deltamethrin compared to those completely lacking the mutations (for example, *CYP6P9a* (SS)/*CYP6P9b* (SS) or *CYP6P9a* (SS)/*CYP6P9b* (SS)/6.5 kb (SS)), as shown in the Table S2. Heterozygote mosquitoes were also more likely to survive exposure to this insecticide than their susceptible homozygote counterparts (Table S2). A significant association was found between the presence of the double and triple homozygote resistant genotypes and the ability of hybrid mosquitoes to survive exposure to 1X, 5X, and 10X deltamethrin, showing that these markers combine to exacerbate the resistance to deltamethrin (Table S2). 

### 3.3. Impact of VGSC Mutations on the Escalation of Permethrin Resistance in An. gambiae

**Figure 5:**
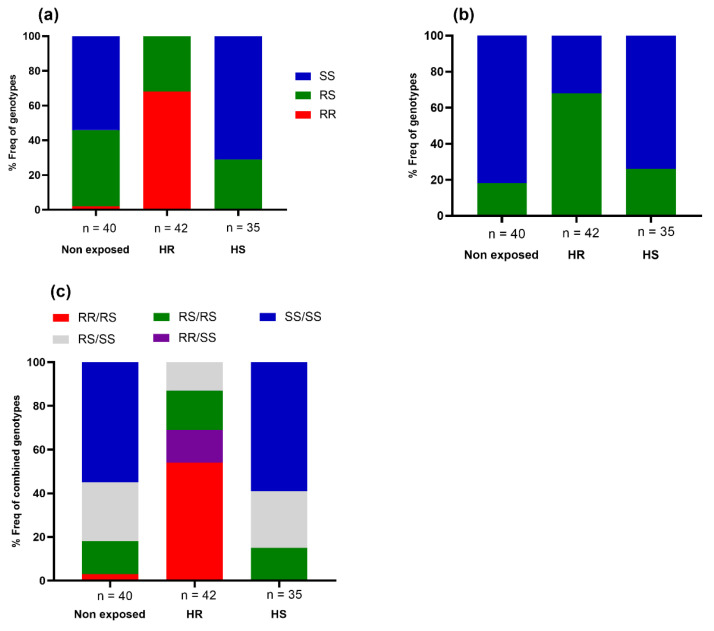
Contribution of VGSC mutations and their combinations in the escalation of pyrethroid resistance in *An. gambiae* hybrid strains. Distribution of genotypes for (**a**) *L1014F*, (**b**) *N1575Y,* and (**c**) a combination of L1014F and N1575Y, between alive and dead after pyrethroid exposure. HR: Alive after 90 min of exposure to permethrin 1X DC (Highly resistant; HS: Dead after 3 min of exposure to permethrin 1X DC (Highly susceptible).

In total, 77 hybrid females (Alive 90 min = 42, Dead 03 min = 35, Non-exposed = 40) were genotyped for the *L1014F* and *N1575Y* mutations in HR and HS after exposure to permethrin 1X. A significant difference in genotype distribution was observed between the HR and HS groups for the *L1014F* (χ^2^: 52.2; *p* < 0.0001) and *N1575Y* (χ^2^: 13.7; *p* = 0.00021) mutations. The homozygote-resistant RR at the L1014F-VGSC locus was only present among the live mosquitoes and was completely absent in the dead group (Figure 5), supporting that this VGSC-resistant marker is associated with permethrin resistance (OR = 2793; *p* = 0.0001; CI = 53.4 to 146,077.1). Regarding the *N1575Y* mutation, homozygote RR was absent, and the frequency of the heterozygote genotype-RS was higher in the survivor group than in the dead group (χ^2^: 13.5; *p* = 0.0002). A greater proportion of the susceptible genotype-SS was in the dead group than in the alive group (Figure 5). A significant association was found between the ability of mosquitoes with the *N1575Y* heterozygote genotype to survive exposure to permethrin 1X compared to those with the susceptible genotype (OR = 6.2; *p* = 0.0004; CI = 2.3 to 16.9). When the two markers were combined, the proportion of RR/RS and RS/RS genotypes was higher in highly resistant than in susceptible mosquitoes (Figure 5). Overall, possessing the two VGSC markers confers a significant ability to survive exposure to permethrin compared to the double susceptible homozygotes (OR = 1763; *p* = 0.0002; CI = 33.3 to 93,077.7) (Table S2).

### 3.4. Contribution of Asaia spp. to the Escalation of Pyrethroid Insecticide Resistance in Malaria Vectors

#### 3.4.1. Prevalence of *Asaia* spp. in *Anopheles* Strains

**Figure 6:**
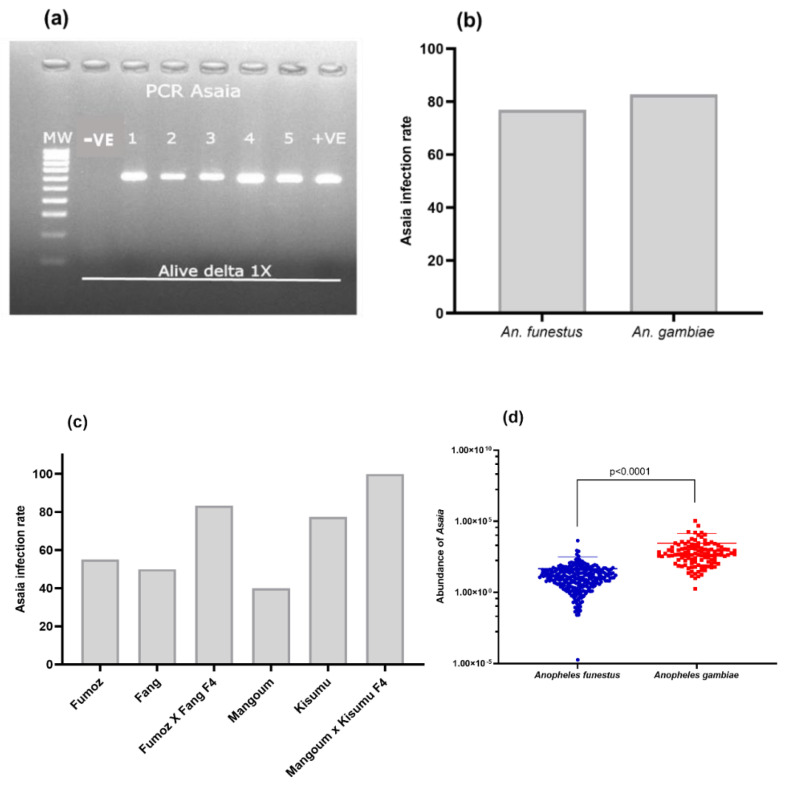
Detection of the *Asaia* spp. symbiont in *Anopheles* samples. (**a**) An electrophoresis gel picture of the PCR products. (**b**) Prevalence of *Asaia* symbiont in *An. funestus* and *An. gambiae* (**c**) Prevalence of *Asaia* in different strains (parental lines and crossings). The prevalence was estimated as the proportion of infected mosquitoes with *Asaia* spp. (**d**) Estimation of the abundance of *Asaia* in overall *An. funestus* and *An. gambiae* mosquitoes tested. The Mann-Whitney test was used to evaluate the differences in *Asaia* loads between the two species.

Overall, *Asaia* spp. was screened in 387 *An. funestus* and 197 *An. gambiae,* including parental and crossing mosquitoes. Prevalence rates of 77% (298/387) and 83.2% (163/196) were recorded in *An. funestus* and *An. gambiae,* respectively (Figure 6). Of the 20 PCR amplicons sent for sequencing, 18 samples with a sequence size of approximately 470 bp were subjected to a BLASTn search in GenBank (NCBI). All sequences were homologous to the reference sequences of *Asaia* spp. No significant difference in *Asaia* prevalence was observed between the two species (χ^2^: 2.9; *p* = 0.08). Focusing on *An. funestus*, the frequency of *Asaia* was almost similar between the laboratory-resistant strain FUMOZ (55%, 22/40) and the reference susceptible colony FANG (50%, 20/40). However, the FUMOZ_X_FANG hybrid line was significantly more infected with *Asaia* spp. (83.4%, 256/307) than FUMOZ (χ^2^: 17.8; *p*< 0.0001) and FANG (χ^2^: 24.1; *p*< 0.0001) (Figure 4). Concerning *An. gambiae* species, the prevalence of *Asaia* was higher in KISUMU (77.5%, 31/40) and the hybrid MANGOUM_X_KISUMU (100%, 116/116), but lower in the MANGOUM field strains (40%, 16/40). As noticed in *An. funestus*, the hybrid strain MANGOUM_X_KISUMU was also significantly more infected with the symbiont when compared to the pure lines MANGOUM (χ^2^: 81.7; *p* < 0.0001) and the susceptible strain KISUMU (χ^2^: 27.5; *p* < 0.0001) (Figure 6).

##### Assessment of the Link between the Prevalence of *Asaia* spp. and Insecticide Resistance in Hybrids FUMOZ_X_FANG

Almost all the alive mosquitoes after exposure to deltamethrin were more infected with *Asaia* spp. with a frequency of 98.5% (136/138), compared to the dead group, which registered a prevalence of 79.7% (110/138) (χ^2^ = 25.5, *p* < 0.0001). This was confirmed by the odds ratio, which showed that mosquitoes infected with *Asaia* had a greater chance of surviving exposure than uninfected mosquitoes (OR = 17.3, *p* = 0.0001, CI = 4 to 74.3). The same pattern was observed with different concentrations of insecticide, where resistant mosquitoes infected with the symbiont were more likely to survive 1X, 5X, and 10X concentrations of deltamethrin compared to the susceptible ones (Table 1). However, a significant association between the prevalence of *Asaia* and the resistance phenotype was found only at 5X concentrations of deltamethrin (OR = 25.8, *p* = 0.002, CI = 3.2 to 203.7).

**Figure 7:**
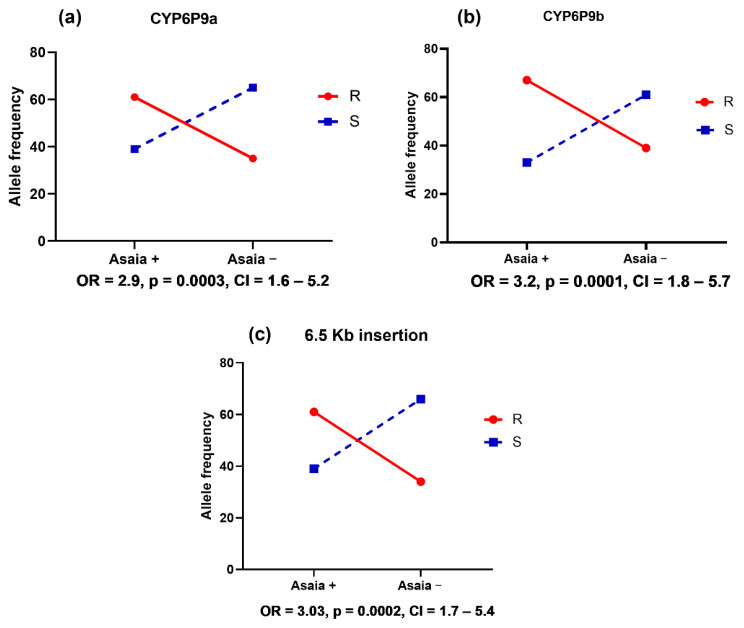
Prevalence of *Asaia* spp. in relation to resistance and susceptible alleles. (**a**) Association between *CYP6P9a*-R and *CYP6P9a*-S alleles and the prevalence of *Asaia* spp. (**b**) Association between *CYP6P9b*-R and *CYP6P9b*-S alleles and the prevalence of *Asaia* spp. (**c**) Association between the alleles of the structural variant *6.5* kb-SV and the prevalence of *Asaia* spp. bacteria.

Considering the genotypes, for all the mutations evaluated, a comparison of the *Asaia* proportion between genotypes showed that homozygous resistant-RR individuals were significantly more infected with the bacteria when compared to the other genotypes. Overall, harboring the resistance alleles *CYP6P9a*_R, *CYP6P9b*_R, and *6.5 kb-SV*_R increased the likelihood of individuals being more infected with the symbiont compared to the susceptible allele (Figure 7). When the markers were evaluated together, the double- and triple-resistant genotypes (RR/RR and RR/RR/RR) were found to be much more infected with *Asaia* spp. compared to the double- and triple-susceptible or heterozygote genotypes. It would also be interesting to highlight an increase in the odds ratio when the markers were combined, thus demonstrating that the presence of more than two homozygous resistant genotypes in mosquitoes would further favour more infection with *Asaia* than with a single allele (Table S3).

##### Association between the Prevalence of *Asaia* spp. and Insecticide Resistance in Hybrids MANGOUM_X_KISUMU

All individuals of this strain (alive and dead) were infected by *Asaia*, hence, no correlation was observed between the phenotype and the infection by the symbiont. Similar to the L1014F and N1575Y VGSC markers, no significant association was found between the marker and *Asaia*.

#### 3.4.2. Abundance of *Asaia* spp. in *Anopheles* Hybrids Strains

The qPCR assay for *Asaia* spp. was performed on 415 *Anopheles* hybrid mosquitoes after exposure to insecticides (*An. funestus* = 307, *An. gambiae* = 116) (Table S4). The relative abundance of *Asaia* spp. in *An. funestus* species ranges from 0.0 to 4392.4 copies, with a median of 11.5 [IQR = 3.2–33.8] and a mean of 47.1. *An. gambiae* crossing, on the other hand, presented a bacterial quantity between 1.8 and 106,619, with a median of 472.5 [IQR = 143.2–1282.4] and a mean of 2849.9. Using the Mann-Whitney test, we observed a significant difference in the abundance of *Asaia* between the two species (*p* < 0.0001) (Figure 6).

##### Assessment of the Link between the Quantity of *Asaia* spp. and Insecticide Resistance in Hybrids FUMOZ_X_FANG

**Figure 8:**
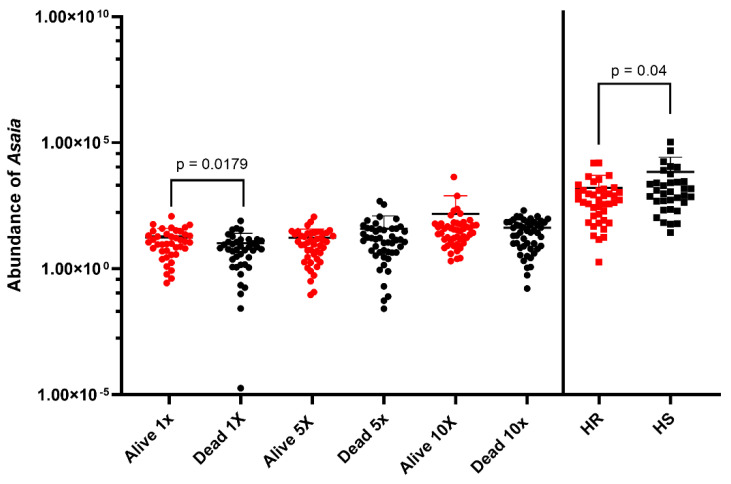
Relationship between the abundance of *Asaia* spp. and insecticide resistance phenotype in hybrids *An. funestus* and *An. gambiae* after exposure to insecticide. HR: Alive mosquitoes after 90 min permethrin exposure to permethrin 1X DC (Highly resistant); HS: Dead 3 min after exposure to permethrin 1X DC (Highly susceptible).

A comparison of the bacterial load between alive (*n* = 135) and dead (*n* = 136) mosquitoes revealed that in resistant individuals, the density varied from 0.1 to 4392, with a median of 15.9 [IQR = 5.6–33.5] and a mean of 64.5. The susceptible group had a load range of 0.0–479.9, a median of 11.2 [IQR = 3.9–38.8], and a mean of 31.5. There was no significant difference in symbiont abundance between the two groups (*p* = 0.3; Mann-Whitney test). Considering the insecticide dose, we observed a significant difference in the amount of *Asaia* between alive and dead mosquitoes only at 1X (*p* = 0.02; Mann-Whitney test) (Figure 8).

The same analysis was performed using the previously identified resistance markers. Using the non-parametric ANOVA test (Kruskal-Wallis test), we found no significant association between any genotype of the different mutations and the abundance of the bacteria (*CYP6P9a*: *p* = 0.4726; *CYP6P9b*: *p* = 0.8231, *6.5 kb-SV*: *p* = 0.3227). The same observation was made for the combined markers (*CYP6P9a/CYP6P9b*: *p* = 0.8384; *CYP6P9a/6.5 kb-SV*: *p* = 0.4912; *CYP6P9b/6.5 kb-SV*: *p* = 0.7517; *CYP6P9a/CYP6P9b/6.5 kb-SV*: *p* = 0.9527).

##### Association between *Asaia* spp. Load and Insecticide Resistance in *Anopheles gambiae* Hybrid Strains

Regarding the bacterial quantity, the *Asaia* load of the HR mosquitoes fluctuated from 1.83 to 15,990, with a median of 533.2 [IQR = 104.6–1264] and a mean of 1632. The HS strain had a bacterial load ranging from 27.3 to 106,619, with a median of 1045 [IQR = 381.6–2739] and a mean of 6.96. Susceptible mosquitoes were thus significantly more loaded with *Asaia* spp. than the resistant group, as shown in Figure 8 (*p* = 0.04; Mann-Whitney test). In addition, we observed that the symbiont was more abundant in the SS and SS/SS genotypes for all the VGSC markers tested compared to other genotypes. However, this was not significant, confirming the negative association between *Asaia* load and permethrin resistance in *An. gambiae* (Table 2).

## 4. Discussion

This study explored the possible association of the *Asaia* symbiont with the observed increased pyrethroid resistance in *An. funestus* and *An. gambiae*. These findings established the contribution of cytochrome P450 markers to the increasing resistance intensity and revealed a contrasting association of the *Asaia* symbiont with insecticide resistance in both *Anopheles* species.

### 4.1. High Level of Resistance to Pyrethroids in An. funestus and An. gambiae Strains

Here, we report a very low mortality rate of FUMOZ mosquitoes against discriminating doses of pyrethroids, showing that the selection for a resistance phenotype with deltamethrin over generations for this reference strain generated a relatively stable resistance to pyrethroids over time [30]. In addition, the results of the WHO bioassays conducted on the hybrid strain *An. funestus* (reared in the absence of insecticide selection) showed an escalation of resistance to deltamethrin. This high level of resistance might be due in part to the combined action of several cytochrome P450-associated loci (rp1) previously identified in the FUMOZ-R strain [41]. Indeed, previous studies have indicated the predominant role of metabolic genes, driven primarily by the duplicated cytochrome P450 genes *CYP6P9a* and *CYP6P9b,* which allow mosquitoes to withstand exposure to pyrethroids [9,42].

While focusing on the field population of *An. gambiae* collected from Mangoum, we registered a high level of resistance to pyrethroids. Similar results were previously obtained in the same locality, revealing a very low mortality rate of mosquitoes to discriminating doses of not only pyrethroids but also carbamates, organophosphates, and organochlorines [32]. This could be explained by the presence of *VGSC* mutation sites imposed by the massive use of insecticides in the locality, probably from both LLINs and agricultural activities, as recently described [32]. The MANGOUM_X_KISUMU mosquito crosses were more susceptible to pyrethroids than the parental lines, exhibiting low resistance intensity. Several cases of reversal insecticide resistance have been reported in *An. gambiae* reared under laboratory conditions [43,44,45]. This indicates that resistance in the field can significantly decrease over time if mosquitoes are not continuously subjected to insecticide pressure [44].

### 4.2. Cytochrome P450 Markers and VGSC Mutations Are Partially Associated with the Escalation of Pyrethroid Resistance in Mosquitoes

Genotyping of *CYP6P9a*, *CYP6P9b*, and *6.5 kb-SV* in *An. funestus* revealed a strong association between these markers and the ability of mosquitoes to survive increasing doses of deltamethrin. This is in line with previous genomic studies, which suggested that metabolic detoxification of cytochrome P450 monooxygenases *(CYP6P9a/b)* appears to be one of the major mechanisms of pyrethroid resistance in both laboratory and field strains of *An. funestus* [35,42,46,47,48,49]. The increased expression of these genes, driven by polymorphisms in the cis-regulatory regions, coupled with allelic variation in the coding region with key amino acid changes [50], would produce stronger metabolic activity against both type I and II pyrethroids. The 6.5-kb SV, on the other hand, has been shown to act as an activator of the expression of the duplicated P450 genes [37]. Indeed, the near fixation of the three genetic elements has been shown to increase metabolic detoxification and escalate the resistance of vectors to pyrethroids [37]. However, their presence could not completely explain the aggravation of deltamethrin resistance observed in this hybrid strain, as our findings demonstrated a decrease in the association between the mutations and the resistance phenotype with high doses of insecticide. Thus, it suggests that other mechanisms besides the metabolic resistance loci could be involved.

Molecular detection of *VGSC* mutations in MANGOUM_X_KISUMU crossed mosquitoes revealed a high frequency of the L1014F resistant allele in highly resistant mosquitoes after 90 min of exposure to permethrin. Indeed, over the past decades, studies have associated this substitution of domain II of the voltage-gated sodium channel with DDT and pyrethroid resistance [51,52,53]. Similarly, a high proportion of the *N1575Y* allele was detected in surviving mosquitoes, further supporting its association with pyrethroid resistance, as it was found to compensate for the deleterious fitness effects of *L1014F* [54]. Nonetheless, these two mutations do not account for all the genetic variance of the super resistance to permethrin detected in this strain, and further research into other factors contributing to the escalation of pyrethroid resistance in this *An. gambiae* strain is required.

The significant correlation observed between both cytochrome P450 and VGSC alleles provided a suitable template for investigating the association between the presence/abundance of specific symbionts and the resistance phenotype and genotype.

### 4.3. Detection of Asaia spp. in Anopheles Strains

Our data revealed that *Asaia* spp. occurred in both *An. funestus* and *An. gambiae* strains, showing that the bacteria is tightly associated with several species of *Anopheles* mosquitoes [55]. This ability to colonize *Anopheles* populations is attributed to its potential for horizontal transmission (through pre-adult and adult mosquito oral feeding), as well as vertical transmission from mother to progeny via ovarian infection to eggs [56,57]. Furthermore, we observed that the offspring of the hybrids (*An. funestus* and *An. gambiae*) were much more infected by the symbiont than the parental lines, thus demonstrating that the crossing carried out would have favored the infection by *Asaia*. This could be explained by the fact that the physiological modifications engendered by the crossing, could have increased the prevalence of *Asaia* to compensate for the demand for essential nutrients and metabolic cofactors (such as carbon, nitrogen, and vitamins) provided by the symbiont [58].

### 4.4. Asaia spp. Is Associated with the Escalation of Pyrethroid Resistance in An. funestus

This study also demonstrated that the prevalence of *Asaia* was significantly higher in FUMOZ_X_FANG resistant hybrids than in susceptible hybrids at each concentration of insecticide tested accompanied by a significant association with the escalation of deltamethrin resistance at 5X. However, this association was reduced at 10X, probably because other factors play a greater role at this resistance level. The presence of the *Asaia* symbiont could alter or reduce the effects of an insecticide in mosquitoes through direct degradation of pyrethroids by the bacterial strain. This was demonstrated in an agricultural pest, the bean bug *Riptortus pedestrians,* for which *Burkholderia* bacteria were able to metabolize fenitrothion, an organophosphate pesticide [22]. Moreover, phylogenomic analysis of *Asaia* strains isolated from *Ceratitis capitata* (fruit fly) led to the detection of an ancestral hydrolase gene that can contribute to the degradation of pyrethroids [25]. Future genomic and transcriptomic studies of the *Asaia* strain from FUMOZ-R could help detect bacterial detoxification genes. Another mechanism through which *Asaia* could contribute to resistance in this strain is by indirectly increasing the expression of multiple detoxification enzymes capable of breaking down pyrethroids. This mechanism was described in *An. stephensi*, where midgut bacteria boost the activity of alpha-esterase and glutathione S-transferases, resulting in temephos resistance [59]. In addition, our findings also showed that homozygote-resistant genotypes for all mutations (*6.5 kb*, *CYP6P9a*, *and CYP6P9b*) or combined mutations were more infected compared to the susceptible genotypes. Despite the lack of information on the association between genetic factors and mosquito microbial variations, we hypothesized that selecting FUMOZ-R mosquitoes for deltamethrin resistance would result in the generation of a parallel resistant strain of *Asaia* to this insecticide, which could be transmitted to the progeny. It is possible that there was a tandem selection of a resistant *Asaia* strain with the cytochrome P450 alleles, which can explain why resistant homozygote genotypes are more infected with the symbiont. Therefore, it will be interesting to isolate and compare the *Asaia* strain from the resistant (FUMOZ) and susceptible (FANG) laboratory strains and field mosquitoes.

Regarding the bacterial load in mosquitoes, the results of this study showed a significant association between the quantity of *Asaia* and the resistant phenotype after exposure to the diagnostic concentration of deltamethrin (1X). This finding supports recent observations that selection pressure leads to the increase of resistance alleles against the effectiveness of insecticides. This could also have an evolutionary impact on the insect microbiota by competing with and altering factors that influence microbial load in the host [21]. Indeed, the abundance of symbionts and the overall composition of the microbiota can rapidly evolve in an insect population in response to natural and artificial selection pressures [21]. This ability has been clearly described in the experimental selection of laboratory strains of the aphid *Aphis gossypii* Glover with spirotetramat, where a significant induction of the relative abundance of several bacterial taxa in their host was detected [60]. Moreover, we also observed that the density/quantity of the Proteobacteria *Asaia* seems to be higher in homozygote-resistant genotypes than in other genotypes for all markers tested, although this was not significant. This is in line with the results obtained by Berticat et al. (2002), who observed that higher densities of *Wolbachia* correlate positively with the presence of insecticide resistance alleles in laboratory and natural populations of *Culex pipiens* mosquitoes [61]. They suggested that mosquitoes may be less efficient in controlling *Wolbachia* density when they carry *ester*- or *ace*-organophosphate resistant alleles because of the physiological cost of resistance. A similar immune deficiency associated with cytochrome P450-based resistance could have occurred and reduced the ability of resistant mosquitoes (FUMOZ_X_FANG) to control the density of *Asaia*.

### 4.5. Asaia Load Is Negatively Associated with the Pyrethroid Resistance in Anopheles gambiae

In contrast to *An. funestus,* all *An. gambiae* mosquitoes were infected by *Asaia,* suggesting that *An. gambiae* is more permissive to the symbiont. This pattern correlates with previous observations on the Afro-tropical vector sowing that *Asaia* was able to greatly colonize several tissues in *An. gambiae* [56]. Since all *An. gambiae* individuals were infected with the bacterium, no association could be established between the prevalence of *Asaia* and the resistance phenotype/genotype in this species.

Nevertheless, in terms of abundance, the quantity of *Asaia* was significantly greater in the susceptible phenotype. This result corroborates a study that suggested that *Asaia* and *Serratia* are overabundant in the susceptible field of *Anopheles coluzzii* mosquitoes exposed to deltamethrin [13]. Although the mechanism behind this phenomenon is not well understood, it could be similar to the case of *Bacillus thuringiensis,* which produces *Sm*Enhancin, which perforates the midgut of *Helicoverpa armigera* [62]. *Asaia* could secrete some substances that increase the permeability of the internal target organs to pyrethroid in *An. gambiae*. Concerning the resistance markers, we observed a high abundance of the bacteria in the homozygous-SS genotype compared to the other genotypes. This pattern was different from that obtained for *An. funestus*, indicating a variable relationship between this symbiont and insecticide resistance in both species. This could be associated not only with the different resistance mechanisms at play, with P450-based metabolic resistance having a different interaction with symbionts than VGSC-based target-site resistance, but also with the different types of insecticides used. Further investigations are needed to understand the contrasting pattern of the effect of *Asaia* on pyrethroid resistance in both species and both resistance mechanisms.

## 5. Conclusions

Our findings demonstrate that in the *An. funestus* strain, the prevalence of the bacteria was mainly associated with the resistant phenotype at 5X and genotype, while for abundance, we detected a difference in the *Asaia* load between alive and dead at 1X. Contrarily, for *An. gambiae*, only the quantification of the bacteria allowed us to visualize that the infection was associated with a susceptible phenotype/genotype. This study provides further evidence for the possible role of *Anopheles* endosymbionts in insecticide resistance. Nevertheless, additional studies are required to better understand how *Asaia* could contribute to insecticide resistance and how this varies between different vector species.

## Figures and Tables

**Table 1:** Association between the prevalence of *Asaia* spp. and the insecticide resistance phenotype in *Anopheles funestus* and *Anopheles gambiae* hybrid strains.

Species	Phenotypes	Tested	Infected	Prevalence of *Asaia* spp.	Comparison of the Prevalence of *Asaia* spp. between Alive and Dead
***An. funestus***	Alive 1X	42	41	97.62	χ^2^ = 4.91, *p* = 0.02; OR = 8.2 *p* = 0.05
	Dead 1X	42	35	83.33	
	Alive 5X	48	47	97.92	χ^2^ = 16.93, *p* < 0.0001; OR = 25.8, *p* = 0.002
	Dead 5X	48	31	64.58	
	Alive 10X	48	48	100	χ^2^ = 4.11, *p* = 0.04; OR = 9.8, *p* = 0.12
	Dead 10X	48	44	91.67	
***An. gambiae***	HR	42	42	100	/
	HS	35	35	100	

OR: Odd ratio, χ^2^ = Chi-square, *p* = *p* value, HR = Highly resistant, HS = Highly susceptible, CI = Confidence interval.

**Table 2:** Association between the abundance of *Asaia* spp. and resistance genotype in *An. gambiae* hybrid strains.

Markers	Genotype	Tested	Median	Mean	Percentiles (IQR)	
					25th	75th
**L1014F**	RR	28	569.16	1832.8	142.19	1205.1
	RS	23	484.32	1457.2	145.24	1970.4
	SS	23	1281.7	9393.1	432.8	5703.5
**N1575Y**	RS	37	587.57	2452	276.09	2199.2
	SS	38	873.93	5570.7	120.4	1784.2
**L1014F and N1575Y**	RR/RS	21	550.76	2103.6	229.51	1074.2
	RS/RS	12	1227.4	2354.1	265.27	4033
	SS/SS	18	1281.7	10,407	195.91	2887

RR = Homozygote resistant genotype, RR = Heterozygote resistant genotype, SS = Susceptible genotype, IQR = Interquartile range.

## Data Availability

All data generated or analysed during this study are included within the article and its additional files.

## Supplementary Materials

The following supporting information can be downloaded at: https://www.mdpi.com/article/10.3390/microorganisms11030644/s1, Table S1: List of primers and probes, Table S2: Association between markers and insecticide resistance, Table S3: Association between prevalence of *Asaia* and genotypes, Table S4: Relative abundance of *Asaia* in *Anopheles* species.
